# Morphologische und funktionelle Diagnostik der koronaren Herzkrankheit mittels Computertomographie

**DOI:** 10.1007/s00059-022-05098-7

**Published:** 2022-03-04

**Authors:** S. Baumann, D. Overhoff, C. Tesche, G. Korosoglou, S. Kelle, M. Nassar, S. J. Buss, F. Andre, M. Renker, U. J. Schoepf, I. Akin, S. Waldeck, S. O. Schoenberg, D. Lossnitzer

**Affiliations:** 1 First Department of Medicine – Cardiology, University Medical Centre Mannheim, Mannheim, Germany and DZHK (German Centre for Cardiovascular Research), partner site Heidelberg/Mannheim, Mannheim, Deutschland; 2 Department for Radiology and Neuroradiology, German Federal Armed Forces Central Hospital Koblenz, Koblenz, Deutschland; 3 Department of Radiology and Nuclear Medicine, University Medical Centre Mannheim, Faculty of Medicine Mannheim, Heidelberg University, Heidelberg, Deutschland; 4 Department of Internal Medicine, Cardiology, St. Johannes Hospital, Dortmund, Deutschland; 5 Department of Cardiology & Vascular Medicine, GRN Hospital Weinheim, Weinheim, Deutschland; 6 Department of Internal Medicine/Cardiology, German Heart Institute Berlin, Berlin, Deutschland; 7 The Radiology Center, Sinsheim, Eberbach, Erbach, Walldorf, Heidelberg, Heidelberg, Deutschland; 8 Department of Cardiology, Angiology and Pneumology, University of Heidelberg, Heidelberg, Deutschland; 9 Department of Cardiology, Kerckhoff Heart Center, Bad Nauheim, Deutschland; 10 Division of Cardiovascular Imaging, Department of Radiology and Radiological Science, Medical University of South Carolina, Charleston, SC USA; 11 Klinik für Kardiologie, Angiologie und Pneumologie, Universitätsklinikum Heidelberg, Im Neuenheimer Feld 410, 69120 Heidelberg, Deutschland

**Keywords:** Koronarstenosen, Kalziumscoring, Kardiale Computertomographie, Koronare Herzkrankheit, Myokardischämie, Coronary stenoses, Calcium scoring, Cardiac computed tomography, Coronarty artery disease, Myocardial ischemia

## Abstract

Die CT(Computertomographie)-Koronarangiographie (cCTA) ist bei Patienten mit niedriger und mittlerer Vortestwahrscheinlichkeit für eine koronare Herzkrankheit (KHK) eine sichere Möglichkeit zum nicht-invasiven Ausschluss signifikanter Koronarstenosen und ermöglicht darüber hinaus auch deren funktionelle und morphologische Beurteilung. Der Stellenwert der cCTA wurde durch die 2019 publizierte ESC(European Society of Cardiology)-Leitlinie zu Diagnose und Management des chronischen Koronarsyndroms gestärkt und hat dadurch eine erhebliche Aufwertung erfahren. Die Bestimmung des Agatston-Scores ist eine klinisch etablierte Methodik zur Quantifizierung des Koronarkalks und hat Einfluss auf die Einleitung einer medikamentösen Therapie. Durch Technologien wie die Einführung der EKG-kontrollierten Dosismodulation und der iterativen Bildrekonstruktion kann die cCTA mit hoher Bildqualität und niedriger Strahlendosis durchgeführt werden. Die alleinige anatomische Darstellung von Koronarstenosen wird derzeit um innovative Techniken wie die myokardiale CT-Perfusion oder CT-FFR (fraktionelle Flussreserve) erweitert, jedoch ist der klinische Stellenwert dieser Methoden noch nicht abschließend geklärt. Die cCTA könnte sich zu einem Weichensteller hinsichtlich der Indikationsstellung für eine invasive Koronardiagnostik/-intervention entwickeln.

## Abstract (en)

Computed tomography coronary angiography (cCTA) is a safe option for the noninvasive exclusion of significant coronary stenoses in patients with a low or moderate pretest probability for coronary artery disease (CAD). Furthermore, it also allows functional and morphological assessment of coronary stenoses. The European Society of Cardiology (ESC) guidelines on the diagnosis and management of chronic coronary syndrome published in 2019 have strengthened the importance of cCTA in this context and for this reason it has experienced a considerable upgrade. The determination of the Agatston score is a clinically established method for quantifying coronary calcification and influences the initiation of drug treatment. With technologies, such as the introduction of electrocardiography (ECG)-controlled dose modulation and iterative image reconstruction, cCTA can be performed with high image quality and low radiation exposure. Anatomic imaging of coronary stenoses alone is currently being augmented by innovative techniques, such as myocardial CT perfusion imaging or CT-fractional flow reserve (FFR) but the clinical value of these methods merits further investigation. The cCTA could therefore develop into a gatekeeper with respect to the indications for invasive coronary diagnostics and interventions.

## Stellenwert der kardialen Computertomographie

Über die vergangenen beiden Jahrzehnte hat die koronare Computertomographie-Angiografie (cCTA) eine rasante Entwicklung durchlaufen. Nach anfänglich rein wissenschaftlicher Anwendung konnte sich die CT dank des technischen Fortschritts als wichtiger Bestandteil der kardialen Diagnostik etablieren.

Die cCTA zeichnet sich neben weiteren Indikationen insbesondere für den Einsatz bei Patientinnen und Patienten mit Verdacht auf das Vorliegen einer koronaren Herzkrankheit (KHK) aus. Die Anwendung bei niedriger bis intermediärer Vortestwahrscheinlichkeit im elektiven und auch akuten Setting stellt ein Haupteinsatzgebiet dieser Untersuchungsmodalität dar [1, 2]. Gemessen an der invasiven Koronarangiographie erreicht die cCTA eine Sensitivität von 95–99 % bei einem negativ-prädiktiven Wert von 97–99 % und ist anderen nicht-invasiven Methoden damit mindestens ebenbürtig [3–6]. Die exzellente diagnostische Genauigkeit geht auf eine hohe räumliche Auflösung und eine insgesamt deutlich verbesserte Bildqualität moderner CT-Systeme zurück. Parallel hierzu ließ sich eine drastische Verringerung der Strahlendosis erreichen [7].

In der vielbeachteten SCOT-HEART-Studie konnte erstmalig eine geringere Rate des kombinierten Endpunkts aus Herzinfarktrate und Gesamtmortalität durch zielgerichtete Therapiesteuerung mittels cCTA bei Patientinnen und Patienten mit stabiler Angina pectoris im Vergleich zur Routinediagnostik gezeigt werden. Vor dem Hintergrund dieser und weiterer Studien sollen im vorliegenden Übersichtsartikel Indikation und Evidenz für den Einsatz der cCTA beleuchtet werden.

## Indikationen nach den aktuellen ESC- und NICE-Leitlinien

Die 2019 publizierte ESC(European Society of Cardiology)-Leitlinie zu Diagnose und Management des chronischen Koronarsyndroms orientiert sich erstmals an der klinischen Präsentation von Patienten mit KHK und unterscheidet zwischen dem akuten (ACS) und dem chronischen (CCS) Koronarsyndrom [1]. Das CCS wird in 6 klinische Szenarien unterteilt, die neben der Angina pectoris auch Dyspnoe als Symptom und mehrere Stadien der vermuteten oder manifesten KHK berücksichtigt. Anhand der Vortestwahrscheinlichkeit werden Patientengruppen definiert, die von einer nicht-invasiven Diagnostik profitieren können (Tab. 1).

**Table 1:** 

	Typisch		Atypisch		Nichtanginös		Dyspnoe^a^	
Alter	M (%)	W (%)	M (%)	W (%)	M (%)	W (%)	M (%)	W (%)
30–39	3	5	4	3	1	1	0	3
40–49	**22**	*10*	*10*	*6*	3	2	*12*	3
50–59	**32**	*13*	**17**	*6*	*11*	3	**20**	*9*
60–69	**44**	**16**	**26**	*11*	**22**	*6*	**27**	*14*
70+	**52**	**27**	**34**	**19**	**24**	*10*	**32**	*12*

Die Felder mit *fettem Text* markieren Patientengruppen, die am meisten von einer nicht-invasiven Diagnostik profitieren (Vortestwahrscheinlichkeit > 15 %); die Felder mit *kursivem Text* beinhalten Patientengruppen mit einer KHK-Vortestwahrscheinlichkeit von 5–15 %, für welche nicht-invasive Diagnostik in Erwägung gezogen werden kann, nachdem die klinische Wahrscheinlichkeit, basierend auf Modifikatoren der Vortestwahrscheinlichkeit, evaluiert wurde

^a^Zusätzlich zu den klassischen Diamond- und Forrester-Klassen werden nun auch Patienten mit Dyspnoe als primärem Symptom einbezogen

**Figure 1:**
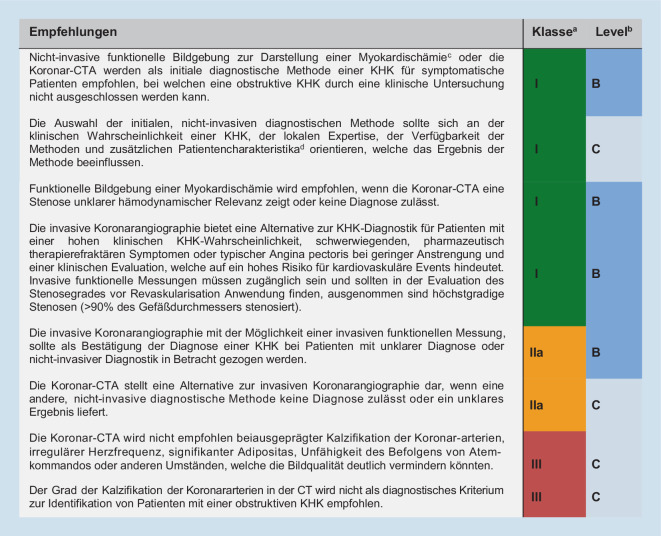

Eine Aufwertung erfuhr die cCTA in diesen Leitlinien (Abb. 1), weil sie erstmals bereits bei asymptomatischen Hochrisikopatienten im Kontext mit funktioneller Bildgebung im Sinne der Risikoevaluation empfohlen wird (Klasse-IIB Empfehlung).

Für symptomatische Patienten, bei denen eine obstruktive KHK durch eine alleinige klinische Beurteilung nicht sicher ausgeschlossen werden kann, wurde die cCTA als initialer Test ebenfalls erstmals gleichwertig mit der nicht-invasiven Ischämietestung (Klasse-IB-Empfehlung) empfohlen. Zudem kann die cCTA bei nicht konklusivem, nicht-invasivem Ischämietest als Alternative zur invasiven Koronarangiographie (Klasse-IIa-Empfehlung) und zum Ausschluss fixierter Stenosen bei V. a. eine vasospastische Angina (Klasse-IC-Empfehlung) erwogen werden. Bevorzugt werden sollte die cCTA bei geringer und mittlerer Vortestwahrscheinlichkeit, bislang fehlender Diagnose einer KHK, dem Wunsch nach Informationen über Ausmaß der KHK und Plaquecharakteristika sowie einer zu erwartenden guten Bildqualität. Berücksichtigt werden müssen hierbei die lokale Verfügbarkeit und Expertise. Eine direkte invasive Koronarangiographie hingegen ist nurmehr bei einer explizit hohen Vortestwahrscheinlichkeit für das Vorliegen einer KHK oder typischer, therapierefraktärer Angina bei geringer Belastungsstufe empfohlen.

Die Leitlinie des National Institute for Health and Care Excellence (NICE) des Vereinigten Königreichs geht über die ESC-Leitlinie hinaus und stellt die cCTA bei neu aufgetretenem Brustschmerz und suspizierter KHK als Methode der ersten Wahl den funktionellen Methoden voran. Auch auf die Berücksichtigung der Prätestwahrscheinlichkeit für eine KHK wird verzichtet [8]. In der Nationalen Versorgungsleitlinie der Bundesrepublik Deutschland hat sich die cCTA bei CCS mit einer Vortestwahrscheinlichkeit von 15–50 % als geeignete diagnostische Methodik etabliert [9].

Im Jahr 2020 empfahlen die NSTEMI(„non ST elevation myocardial infarction“)-Leitlinien der ESC die cCTA erstmals als Alternative zur invasiven Koronarangiographie zum Ausschluss eines ACS bei Patienten mit einer geringen bis mittleren Vortestwahrscheinlichkeit für eine KHK, normalen Troponinwerten und unauffälligem oder nichtkonklusivem EKG (Klasse-IA-Empfehlung; [2]). CT-FFR (fraktionierte Flussreserve) hatte hierbei noch keine Berücksichtigung gefunden.

Nicht empfohlen hingegen wird die cCTA für Follow-up-Untersuchungen, da in der klinischen Routine derzeit nur begrenzt Aussagen zur hämodynamischen Relevanz von Koronarstenosen getroffen werden können. Die sehr umfangreichen Leitlinien der Society of Cardiovascular Computed Tomography (SCCT) bieten eine noch umfassendere und detailliertere Beschreibung der Durchführung und Befundung von cCTA an [10].

## Bildakquisition in der kardialen Computertomographie

**Figure 2:**
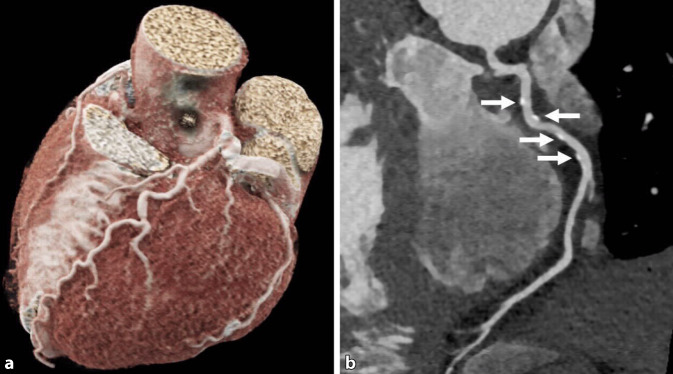

Die Analyse der Koronararterien kann mit 2 sich ergänzenden Techniken erfolgen: zum einen mit der nativen CT zur Darstellung und Quantifizierung des Koronarkalks (Agatston-Score [Kalziumscoring]) und zum anderen mit der kontrastmittelgestützten cCTA (Abb. 2). Die Akquisition der kardialen cCTA erfolgt EKG-gesteuert. Hierzu gibt es 3 Möglichkeiten, die unterschiedliche Vor- und Nachteile bieten.

Die heute am weitesten verbreitete Methode ist die prospektive EKG-Triggerung, bei der zu einer *a priori* festgelegten Herzzyklusphase die Aufnahme des Herzvolumens erfolgt. Da nur bei wenigen CT-Scannern mit entsprechend großer Detektorbreite die Abdeckung des gesamten kardialen Volumens in einer Teil- oder Vollrotation erfolgen kann, werden zumeist mehrere sich direkt anschließende Aufnahmen sequenziell entlang der z‑Achse akquiriert, die in der Nachverarbeitung zusammengefügt werden („step and shoot“).

Die Akquisition des CT-Datensatzes bei der prospektiven EKG-Triggerung erfolgt üblicherweise in der Enddiastole. Eine Darstellung der Koronararterien in der Phase der isovolumetrischen Relaxation der Systole ist Sonderfällen wie Vorhofflimmern, erhöhten Herzfrequenzen und der TAVI(„transcatheter aortic valve implantation“)-Planung vorbehalten [11–13].

Die zweite Möglichkeit der Akquisition der cCTA ist das retrospektive EKG-Gating. Hierbei wird das Herzvolumen durch eine Spiralabtastung für den gesamten Herzzyklus erfasst und dem EKG nach dem CT-Scan retrospektiv zugeordnet. Der große Vorteil dieser Methode ist ihre Robustheit, da der gesamte Herzzyklus zur Beurteilung der Koronararterien herangezogen werden kann. Des Weiteren können zusätzliche Informationen wie Herzvolumina, Wandbewegung des Myokards sowie die funktionelle Darstellung der Herzklappen ausgewertet werden. Der Nachteil des retrospektiven EKG-Gatings ist die höhere Strahlendosis der Untersuchung im Vergleich zur prospektiven Aufnahmetechnik. In den letzten Jahren konnte durch neue Detektorgenerationen und hierdurch ermöglichte Niedrig-kV-Untersuchung neue Rekonstruktionsalgorithmen (iterative Rekonstruktion) und Modulation des Röhrenstroms während der Herzzyklusphasen, also Absenkung des Röhrenstroms in Bereichen des Herzzyklus ohne erhöhte diagnostische Wertigkeit, die Strahlenexposition signifikant auch für das retrospektive EKG-Gating gesenkt werden.

Die dritte Technik zur Darstellung der Koronararterien mittels CT ist die High-pitch-Spirale, bei der mittels sehr schnellen Tischvorschubs die Darstellung der Koronararterien als extrem schnelle CTA erfolgt, mit dem Unterschied des EKG-getriggerten Starts der Untersuchung im Gegensatz zu einer regulären CTA. Dem Vorteil der geringen Strahlendosis dieser Untersuchungstechnik steht der Nachteil der apparativen Verfügbarkeit sowie der verminderten Robustheit der Aufnahmetechnik entgegen, sodass dieses Verfahren Patienten mit einer niedrigen rhythmischen Herzfrequenz vorbehalten ist.

Festzuhalten ist, dass es über alle Methoden der cCTA-Akquisition hinweg im letzten Jahrzehnt zu deutlichen Dosisreduktionen gekommen ist, wie die PROTECTION-VI-Studie zeigen konnte. Gemittelt liegt das Dosislängenprodukt einer cCTA hier bei 195 mGy*cm, was je nach angewendetem Konversionsfaktor 2,7 mSv (Konversionsfaktor: 0,014 mSv/mGy*cm) oder 5,1 mSv (Konversionsfaktor: 0,026 mSv/mGy*cm) entspricht [7]. Jedoch ist es mit aktuellen CT-Scanner-Generationen bereits möglich, cCTA-Untersuchungen mit einer Dosis von weniger als 1 mSv durchzuführen [14].

Ebenfalls immer mehr an Gewicht gewinnt die strukturierte Befundung. In diesem Zusammenhang sollte zur Beurteilung einer cCTA das CAD-RADS™ (Coronary Artery Disease Reporting and Data System) verwendet werden (Tab. 2), da hierdurch eine interdisziplinär einheitliche Sprache der CT-Befundung implementiert wird [15].

**Table 2:** 

CAD-RADS 0	Keine sichtbare Stenose (0 %)
CAD-RADS 1	Minimale Stenose (1–24 %)
CAD-RADS 2	Milde Stenose (25–49 %)
CAD-RADS 3	Moderate Stenose (50–69 %)
CAD-RADS 4	Schwere Stenose (70–99 %)
CAD-RADS 5	Totaler Gefäßverschluss (100 %)

**Figure 3:**
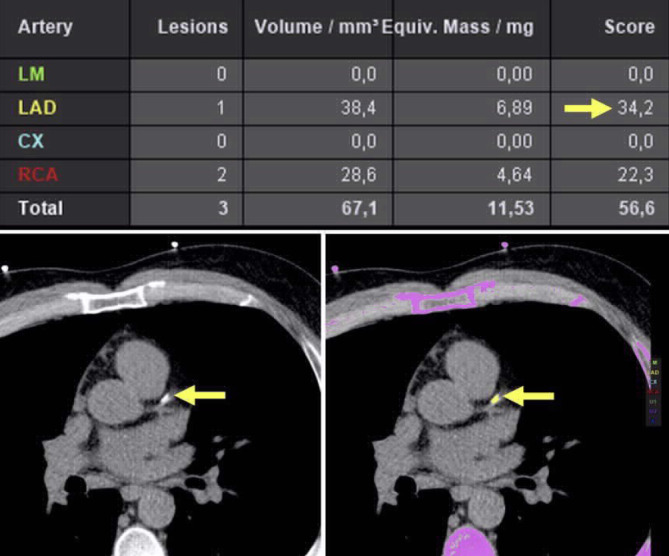

Die eingangs erwähnte weitere Technik zur Beurteilung der Koronararterien ist das Kalziumscoring, welches nach der Agatston-Methode erfolgt. Prinzipiell wird ein nativer CT-Datensatz mit meistens 120 kV in 3 mm Schichtdicke prospektiv EKG-getriggert oder retrospektiv EKG-synchronisiert akquiriert [17]. Um den Agatston-Score zu berechnen, werden alle kalzifizierten Areale mit einer Dichte von mehr als 130 Hounsfield-Einheiten („Hounsfield units“ [HU]) eingeschlossen, die mindestens 1 mm^2^ umfassen. Je nach maximaler HU-Dichte der Plaque wird ein Wert von 1 bis 4 vergeben, der mit der jeweiligen kalzifizierten Fläche multipliziert wird. Die Gesamtsumme aller Plaques des Koronarbaums ergibt den Agatston-Score. Der Score spiegelt somit nicht die eigentliche Masse oder das Volumen der kalzifizierten Areale wider, hat sich aber entgegen den beiden genannten Werten in der klinischen Risikostratifizierung (Tab. 3) durchgesetzt (Abb. 3). Neuere Akquisitionsmethoden erlauben durch Abweichungen von der ursprünglichen Konvention zu niedrigeren Röhrenspannungen (kV) sowie Kombination mit einer High-pitch-Spirale eine Reduktion der Strahlendosis bei guter Korrelation zur ursprünglichen Agatston-Methode [18, 19].

**Table 3:** 

Kalziumscore	Therapie
0	Keine Statintherapie und erneute Reevaluation in 5 bis 10 Jahren, solange keine anderen Risikofaktoren (Diabetes mellitus, positive Familienanamnese, Nikotinabusus) vorliegen
1–99	Einleitung einer Statintherapie bei Patienten älter als 55 Jahre
> 100 oder über der 75. Perzentile	Einleitung einer Statintherapie

## Hochrisikoplaques und CT-Koronarangiographie als prognostische Langzeitparameter

**Figure 4:**
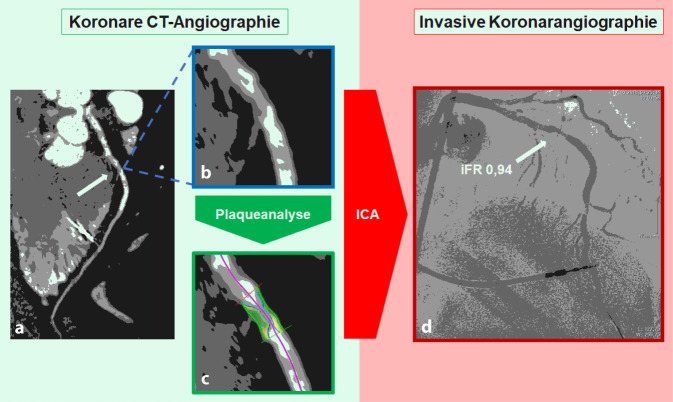

Die cCTA stellt durch den Einsatz zusätzlicher Softwareapplikationen und die Kombination aus funktioneller und morphologischer Plaquequantifizierung einen enormen Fortschritt in der Beurteilung der Plaquekomposition und der hämodynamischen Relevanz von Koronarläsionen sowie der Indikationsstellung von invasiven Koronarangiographien dar. V. a. durch ihren hohen negativ-prädiktiven Wert eignet sich die cCTA zum sicheren Ausschluss einer obstruktiven bzw. stenosierenden KHK [21]. Neben der alleinigen konventionellen cCTA mit visueller Beurteilung von Koronarläsionen durch den Untersucher können zusätzlich Plaquemerkmale wie der koronarangiographische Index auf Poiseuille-Basis (Läsionslänge/minimaler Lumendurchmesser^4^), die minimale Lumenfläche, der minimale Durchmesser und der Prozentsatz des aggregierten Plaquevolumens sowie funktionelle Plaquemarker wie der korrigierte Dichtegradient („corrected coronary opacification“ [CCO]) und der Remodelling-Index (RI) den Nachweis von hämodynamisch signifikanten Koronarstenosen verbessern [22]. Zusätzlich zur bisherigen klassischen anatomischen Stenosegraduierung können mithilfe der cCTA und einer semiautomatischen Softwareapplikation Koronarplaques anhand unterschiedlicher CT-Dichtewerte der Plaquebestandteile hinsichtlich ihrer Zusammensetzung analysiert werden ([23]; Abb. 4). Die Plaquebeschaffenheit kann sowohl Hinweise zur hämodynamischen Relevanz von Koronarläsionen als auch prognostische Informationen im Hinblick auf die Plaquestabilität liefern. Die sog. Low-attenuation-Plaques, bestehend aus einem weichen, lipidreichen Plaquekern mit niedrigen CT-Dichte-Werten, sowie „spotty calcifications“ und weiche Plaques, umgeben von einer dichteangehobenen Hülle („napkin ring sign“), werden als instabile Hochrisikoplaques eingestuft. Des Weiteren gehören Koronarläsionen mit einem positiven RI von mehr als 1,1, welcher einen reaktiven Umbauvorgang in den Koronargefäßen beschreibt, zu den vulnerablen Plaques, die mit einem vermehrten Auftreten kardiovaskulärer Ereignisse assoziiert sind [24, 25]. Die Einleitung einer Statintherapie erfolgt in Abhängigkeit von Kalziumscore, Alter und zusätzlich bestehender kardiovaskulärer Risikofaktoren (Tab. 3; [20]).

## Myokardperfusions-CT

**Figure 5:**
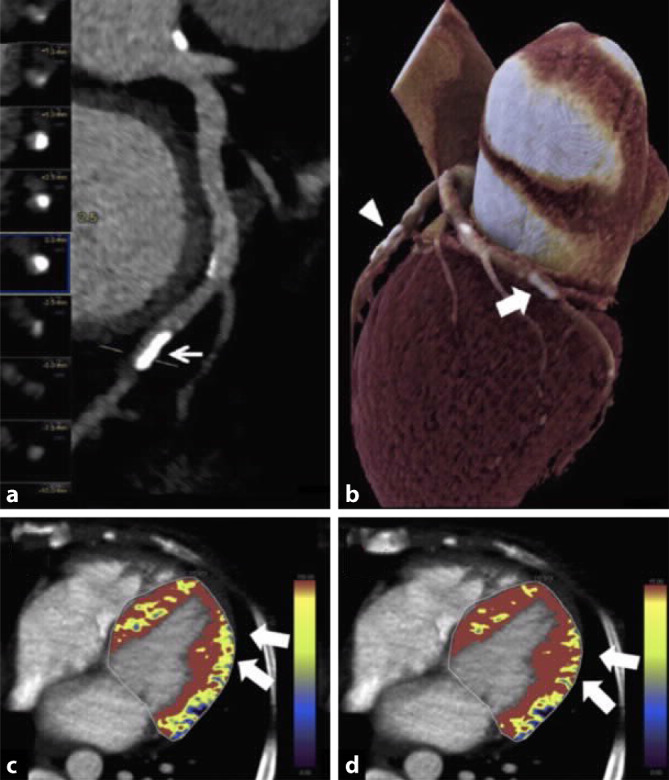

Die myokardiale CT-Perfusion wurde Anfang der 2010er-Jahre als neue Methode zur Evaluation einer myokardialen Ischämie eingeführt [26]. Diese Modalität kombiniert die anatomische Darstellung der Koronararterien mit Messungen der myokardialen Perfusion, um die hämodynamische Relevanz von Koronarstenosen besser beurteilen zu können. Die CT-Perfusion ermöglich hierbei durch die Visualisierung einer verminderten oder verzögerten Kontrastmittelaufnahme des Myokards eine quantitative wie auch qualitative Beurteilung der Myokardperfusion [27]. Ähnlich der Ischämiediagnostik per Stressmagnetresonanztomographie wird eine Bildakquisition sowohl unter Ruhebedingungen als auch unter pharmakologischer Belastung durchgeführt, um zwischen fixierter und induzierbarer Myokardischämie differenzieren zu können. Die technischen Möglichkeiten der Bildakquisition können in statische und dynamische Perfusionsbildgebung unterschieden werden. Die statische CT-Perfusion wird unter Zuhilfenahme von CTA-Datensätzen zum Zeitpunkt der höchsten Kontrastmittelaufnahme anhand eines „snapshot“ berechnet und erfordert somit keine zusätzliche Bildakquisition. Mithilfe dieser Technik kann nur eine qualitative Beurteilung der Myokardperfusion erfolgen, da keine eigentliche Passage des Kontrastmittels durch das Myokard aufgezeichnet wird. Demgegenüber erlaubt die dynamische CT-Perfusion eine reale Beurteilung der Myokardperfusion, indem nach Kontrastmittelinjektion in kurzer Abfolge multiple Bildsequenzen aufgezeichnet werden [28]. Auf Basis dieser Bildakquisition lassen sich Zeit-Dichte-Kurven für alle Bereiche des Myokards erstellen, welche eine quantitative und semiquantitative Beurteilung der Myokardperfusion ermöglichen. Da die Bildakquise der dynamischen CT-Perfusion keine Beurteilung der Koronargefäße gewährleistet, ist eine zusätzliche konventionelle CTA notwendig, was mit einer zusätzlichen Strahlenexposition und Kontrastmittelgabe für den Patienten verbunden ist (Abb. 5). Die diagnostische Genauigkeit der statischen und der dynamischen CT-Perfusion liegt bei einer Sensitivität von 89 % bzw. 88 % und einer Spezifizität von 77 % bzw. 89 % [29]. Die CT-Perfusion ermöglicht somit eine hohe diagnostische Aussagekraft bezüglich einer myokardialen Ischämie, jedoch noch zum Preis einer relativ hohen Strahlendosis von etwa 10–12 mSv (dynamische CT-Perfusion). Dieser Umstand und die relative Komplexität der Untersuchung haben jedoch eine klinische Etablierung und routinemäßige Anwendung bislang in westlichen Ländern verhindert, sodass diese Bildgebungsmodalität aktuell nur im Rahmen von klinischen Studien getestet wird. Jedoch scheint sich die Methode im asiatischen Raum zu einem klinisch breit angewandten Test zu entwickeln.

## CT-basierte Messung der fraktionellen Flussreserve

**Figure 6:**
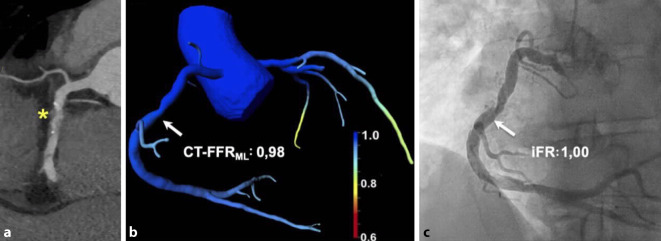

Die FFR-Messung ist der Goldstandard zur invasiven Bestimmung der hämodynamischen Relevanz von Stenosen in den Herzkranzgefäßen. Daneben konnte sich seit 2018 die iFR (*„*instantaneous wave*-*free ratio“) mit Verzicht auf die Gabe von Adenosin als eine gleichwertige Klasse-IA-Empfehlung in den Leitlinien zur myokardialen Revaskularisation der ESC etablieren [30]. In Anlehnung an den invasiven Goldstandard der FFR-Messung konnte durch den Fortschritt in der kardialen CT-Technik die FFR-Messung, basierend auf der Computertomographie (CT-FFR), großes Interesse für die nicht-invasive hämodynamische Bewertung von Stenosen bei Patienten mit KHK wecken (Abb. 6; [31–33]). Schon die ersten Studien zeigen das Potenzial der CT-FFR im Vergleich zum etablierten invasiven Goldstandard. Darüber hinaus weist diese Technik in den großen multizentrischen Studien eine starke Korrelation mit der invasiven FFR auf [34–36]. Die Studien demonstrieren, dass durch die Hinzunahme der CT-FFR im Vergleich zur alleinigen CT-morphologischen Beurteilung von Stenosen die diagnostische Genauigkeit hinsichtlich der hämodynamischen Relevanz von Koronastenosen signifikant verbessert werden kann. Douglas et al. konnten in der veröffentlichten PLATFORM-Studie eine signifikante Reduktion rein diagnostischer invasiver Koronarangiographien durch den vorangestellten Einsatz der CT-FFR in Kombination mit der koronaren CT feststellen [37]. Zu Beginn basierte die verwendete CT-FFR-Technik noch auf einem Algorithmus, der zur Berechnung der Hämodynamik auf computergestützte flussdynamische Modelle zurückgreifen musste. Als Beispiel ist hier der durch die Food an Drug Administration (FDA) im Jahr 2015 zugelassene CT-FFR-Algorithmus FFR_CT_ von HeartFlow Inc. (Redwood City, CA, USA) zu nennen. Entscheidender Nachteil dieses Algorithmus war der große Rechenaufwand, der nur durch den Datentransfer an externe Rechenzentren zu bewältigen war. Abhilfe konnten Weiterentwicklungen der CT-FFR-Algorithmen schaffen, die eine Berechnung vor Ort ermöglichten. Außerdem konnte unter Zuhilfenahme der künstlichen Intelligenz im Sinne eines Machine-Learning-Algorithmus (CT-FFR_ML_) die Kalkulationszeit weiter reduziert und somit bei gleichbleibender diagnostischer Leistung eine intrahospitale, klinische Anwendbarkeit ermöglicht werden [38]. Erwähnenswert ist, dass die CT-FFR-Algorithmen bislang nur mit der FFR als Referenzstandard verglichen wurden. Eine im Jahr 2019 veröffentlichte klinische Arbeit untersuchte die koronare CT-Technik in Kombination mit der CT-FFR_ML_ im Vergleich zur iFR als weiteren invasiven Goldstandard. Hierbei konnte mithilfe der auf einem Machine-Learning-Algorithmus basierten CT-FFR sowohl auf Ebene der klinischen Praktikabilität als auch auf Ebene der diagnostischen Genauigkeit zum einen eine gute intrahospitale Anwendbarkeit gezeigt und zum anderen eine hohe diagnostische Genauigkeit im Vergleich zur iFR festgestellt werden [39]. Diese funktionelle nicht-invasive Bildgebungsmodalität kann dazu beitragen, dass die koronare CT als Weichensteller eine entscheidende Rolle in der Diagnostik der KHK einnimmt [36].

## Fazit für die Praxis

- Die koronare Computertomographie-Angiographie (cCTA) ist bei geeigneten Patienten eine sichere Möglichkeit zum Ausschluss signifikanter Koronarstenosen.
- Durch neuartige Technologien kann die cCTA mit hoher Bildqualität und niedriger Strahlendosis durchgeführt werden.
- Die Bedeutung der cCTA wird in den aktuellen ESC(European Society of Cardiology)-Leitlinien gestärkt und erfährt eine wesentliche Aufwertung.
- Kalziumscoring ermöglicht eine gute Risikostratifizierung mit positivem Einfluss auf die Einleitung einer medikamentösen Therapie.
- Innovative Techniken wie die myokardiale CT-Perfusion oder die CT-FFR (fraktionelle Flussreserve) verbessern die diagnostische Genauigkeit, jedoch ist der klinische Stellenwert noch nicht gänzlich geklärt.
